# Gut thinking: the gut microbiome and mental health beyond the head

**DOI:** 10.1080/16512235.2018.1548250

**Published:** 2018-11-30

**Authors:** Grace Lucas

**Affiliations:** School of Health Sciences, City, University of London, London, UK

**Keywords:** Mental health, gut microbiome, gut microbiota, microbiome–gut–brain axis, anxiety, depression, embodiment, medical humanities

## Abstract

**Background:** In recent decades, dominant models of mental illness have become increasingly focused on the head, with mental disorders being figured as brain disorders. However, research into the active role that the microbiome-gut-brain axis plays in affecting mood and behaviour may lead to the conclusion that mental health is more than an internalised problem of individual brains.

**Objective:** This article explores the implications of shifting understandings about mental health that have come about through research into links between the gut microbiome and mental health problems such as depression and anxiety. It aims to analyse the different ways that the lines between mind and body and mental and physical health are re-shaped by this research, which is starting to inform clinical and public understanding.

**Design:** As mental health has become a pressing issue of political and public concern it has become increasingly constructed in socio-cultural and personal terms beyond clinical spaces, requiring a conceptual response that exceeds biomedical inquiry. This article argues that an interdisciplinary critical medical humanities approach is well positioned to analyse the impact of microbiome-gut-brain research on conceptions of mind.

**Results:** The entanglement of mind and matter evinced by microbiome-gut-brain axis research potentially provides a different way to conceptualise the physical and social concomitants of mental distress.

**Conclusion:** Mental health is not narrowly located in the head but is assimilated by the physical body and intermingled with the natural world, requiring different methods of research to unfold the meanings and implications of gut thinking for conceptions of human selfhood.

## Background

### Gut thinking

Diagnosable mental health problems are said to affect one in four people in any given year [1], they are a leading source of disability globally, and new strategies for prevention and treatment are vital [2]. But the boundaries of these problems sit on shifting sands. Since the anti-psychiatry movement of the 1960s [3] and the analysis from critical psychiatry that has followed [4], debates have continued about what is constitutive of mental illness or mental disorder, especially given the changing nature of psychiatric diagnostic manuals over time [5]. Neuro-explanations of mental health have dominated the last decades of mental health research with mental disorders being re-cast as brain disorders [6], but often on shaky evidential ground and widely contested, especially by social psychiatrists [7].

Whilst there has been disagreement and controversy over the past century, mental health has predominantly been understood as related to the head. Biological psychiatry’s paradigm of brain disorder provides mental illness with a clear physicalized location there [6], whilst a Cartesian dualist model of the immaterial mind contrasts the intangible realm of mental thoughts with the body and physical health [8]. If the body below the head has been involved in understanding mental health, it has often been viewed as a ‘dustbin’ expressing symptoms of illness from above [9]. However, in recent years, research into the microbiome–gut–brain axis has foregrounded the impact of the gut microbiome on mental health [10], inverting these dominant top-down models of mental illness. This emerging evidence, which shows observable links between gut dysbiosis and some mental health conditions, suggests that mental health is not all about the head, after all, leading to some paradigm shifting interpretations and conclusions about what is actually meant by ‘mental’ health, and how it should be treated [12].

Evidence connecting microbiome-gut-brain communication to psychiatric illness might seem to relegate dualistic mind-body thinking to the past, but a Cartesian divide between mental and physical health runs down the middle of healthcare systems (the NHS in the UK is divided in this way despite an increasing focus on the provision of integrated care) [13]. This divide is underpinned by language, which both supports and constructs clinical and non-specialist understandings of the mind and the mental as separate from the body and the physical. As psychiatrist Edward Bullmore argues, a Cartesian ‘blind spot’ [14] intervenes every time mental health is redefined as our language separates out the mental from physical life.

Whilst research into the microbiome–gut–brain axis turns its attention from the brain to the gut and back again, it arguably lacks the conceptual tools to investigate the broader (social, cultural, linguistic) implications of these shifts on the meaning of mental health. In particular, there is a critical imperative in analysing how language is employed. This is because psychiatric diagnostics frame mental health problems, and psychiatry’s vocabularies and definitions are passed onto patients. As philosopher Havi Carel argues, patients are then ‘quick to mimic the medical discourse’ as they re-articulate and explain their conditions to others [15]. Mental health is thus shaped and defined not only within medical settings but also outside of the clinic. The deinstitutionalisation of mental health problems like depression, as argued by feminist scholar Elizabeth A. Wilson, means that they are now ‘extensively entangled with everyday life’ requiring an analysis that extends beyond biomedicine to examine social, cultural and individual constructs [16] (p. 10). Brain or neuro-explanations for mental disorders have disseminated rapidly into popular understanding [17]. Indeed, it is the dominant frameworks of what physical cultures scholar, Simone Fullagar describes as, ‘neuroscience and psy-expertise’ [18] (p. 42) that have provided a structure through which those with mental health problems have sought to understand their experiences in recent decades.

Interest in the microbiome–gut–brain axis has already led to the publication of a range of popular books on the topic [19–21] which argue, in different ways, how communication between the brain, gut, and microbiome may be responsible for a range of health problems including depression and anxiety thereby challenging brain-shaped understandings of mental health. Indeed, if, as Wilson argues, the gut is ‘an organ of mind’ (p. 5) [16] then critical questions are raised: What and where exactly is our ‘mental’ health? How can this research contribute to an improved understanding of selfhood beyond the head? What kind of research is needed to investigate the broader meanings and implications for understanding human mental and physical life?

In this paper, I argue that an interdisciplinary critical medical humanities framework [22] can attend to such ‘ontological questions’ [23] raised by this idea of the gut as an active part of the mind. I take a feminist new materialist approach to argue that – as feminist theorist Karen Barad writes – ‘matter matters’ and that human subjectivity is always ‘entangled’ in the world [24], in opposition to models that theorise the power of ‘mind over body’ [25]. I propose that knowledge about the gut microbiome’s effect on behaviour complicates understandings of mental life and selfhood as contained within the narrating, thinking brain. I build on research into disciplinary entanglements developed in medical humanities scholarship [26] to help contextualise the broad impact of questions around the mind and mental health, calling attention to the way in which research into the microbiome–gut–brain axis illuminates the interplay between biological, psychological and social constructions of mental illness. Finally, I use this critical framework to ask how research into the gut microbiome relates to understandings about selfhood, the way matter is conceptualized, and how embodiment is theorised.

## Mental health is in the head

### All in the mind

Dualism, in philosophy of mind, is the theory that the mind or the mental is different to the body and the physical. This theory has lineage in ancient philosophical thought. Plato thought that the soul, although united to the body, existed beyond it [27]. Philosopher, Rene Descartes (1596–1650) like Plato, also held to these beliefs, but his dualism specifically separated out the ‘mind’ from the body [28]. Descartes’ shaping of the palpable physical body as separable from the intangible mind laid the ground for medicine to claim the physical body as its domain [29]. By the nineteenth century – as psychiatrist R.E. Kendell argues – Cartesian thinking was supported by findings from medical dissection, which showed that patients diagnosed with forms of madness did not show ‘the obvious pathological changes’ that were physically located in other diseases [30]. However, this period saw a diverse range of opinions about the origins of mental illness and – as psychiatry as a discipline began to evolve – other traditions linking the gut and the mind in more holistic conceptions of mind-body interactions were also being re-explored and studied [31]. Indeed, as historian Elizabeth A. Williams argues, the work of the founder of French psychiatry, Philippe Pinel, considered ‘mental and nervous ills’ to be ‘inextricable blends of physical and moral components’ influencing how French physicians approached neuroses and some cases of mental illness [32]. But, as the nineteenth century wore on, some of the theories on the links between gastric and mental illnesses were rejected [33], emerging materialist psychiatric models, which posited that mental illnesses were brain-based ‘cerebral illnesses’ [34] were also challenged [35] and Cartesian influences took hold. Functional illnesses, as opposed to organic disorders, were thought of as being ‘all in the mind’ and therefore not real [36]. Psychogenic (non-physical, non-substance-like) causes for mental disorders underpinned influential theories and techniques of psychoanalysis and psychiatric models into the twentieth century.

### Mental disorders: brain disorders

Although Cartesian understanding set the language of mental against physical illness, in recent decades, images of the brain have been used to depict mental health, bringing a physical location back to understandings of the mind. As Nikolas Rose and Joelle M. Abi-Rached argue – in their critical examination of the ‘new brain sciences’ – for a public highly engaged with brain culture, ‘mind seems visible in the brain itself’ [17] (p. 5). The American National Institute of Mental Health (NIMH) has stated that ‘mental disorders are disorders of the brain’ [6] and The Brain & Behavior Research Foundation uses the term ‘brain and behavior disorders’, arguably taking the word ‘mental’ out of the equation [37].

### All in the mind or brain: the problems

Whether mental disorders are brain illnesses or a matter of immaterial thoughts, there has been a clear focus for mental health as conceptualised within the head. Two recently launched UK charities designed to support mental health, Heads Together [38] and Headcase [39], and the meditation mental health app, Headspace [40], underline this commonly articulated emphasis. Indeed, ‘headclutcher’ imagery, depicting a person holding their head in their hands, is commonly used to accompany articles about mental illness [41].

If medical and public understanding predominantly locates mental health in the head, a question may follow about why this is a problem. Mental health is certainly at a critical point (in terms of economic, societal, and personal costs) and existing research has struggled to move forward the field [42]. Dualist formulations of mental health separate people into parts and prop up stigmatising beliefs about mental health as an individual’s fault – either seen as not real or possible to control from above by thought management. Furthermore, they complicate understanding of the profound physical effects of mental health issues. Indeed, whilst depression and anxiety are defined as mental health problems, the palpable physical symptoms attached to them uncomfortably cross the medical divide [43]. Brain disorder models are equally problematic, as philosopher Natalie F. Banner argues, they operate at a reductionist ‘biological, subperson’ level and often exclude broad-based social, emotional and psychological factors creating internalised models of mental illness [36].

## Getting out of the head: guts and mental health

Research into the gut microbiome has started to unsettle the narrow focus for mental health above the neckline. The observed links between disturbance of the gut microbiome (dysbiosis) and stress, anxiety, and depression have shifted the research ground for mental disorder [12,44,45]. In response to these emerging findings, probiotics and dietary approaches have been explored in terms of their ability to modulate microbiota and address symptoms [46]. Arguably, gut microbiome research starts to move mental health out of the rule of the cognitive head and provides evidence that the gut is storing, remembering, feeling and thinking in itself. However, how this research speaks to the questions around definitions of mind and the mental needs to be addressed.

In examining how the gut microbiome might affect states of mind in relation to the biology of microbiome–gut–brain communication, a question is raised as to what understanding of mental health and minded states is being foregrounded. If microbiome–gut–brain axis research is in alignment with an expanded brain disorder model of mental health – including the assumptions that go along with that model (mental states are brain states) – then the same social psychiatry counter arguments around the displacement of psycho-social human reasons in understanding mental health may also hold true. Furthermore, if it is under the individual’s control to manage their microbiome, and this is what affects mood and mental health, then the implication is that it is the job of the individual to fix it when that mood is low. Indeed, mainstream publishers have been keen to emphasise this particular facet of dietary change in gut health, building on the multi-billion dollar diet industry to sell books from early scientific findings [47,48]. The idea that some people will be enabled to beat or outwit gut bacteria with the right access to the right foods or supplements, feeds into a new gut consumer culture wherein some people will be enabled and others disadvantaged. Recovery solutions move sideways from the self-management therapeutic strategies of the cognitive model to ones based in the gut. These potential alignments with existing understandings of mental wellbeing require careful analysis and exploration.

## A critical medical humanities intervention

### Disciplinary entanglements

The questions pertaining to the shifting boundaries of mind and the mental which are raised by evidence about the gut microbiota on mental health cannot be solved by biomedicine alone. In setting out the project of a critical medical humanities focused on disciplinary entanglement rather than the integration of separate silos of investigation, Des Fitzgerald and Felicity Callard suggest that it is necessary to, ‘understand how practices of making, breaking and shifting boundaries *constitute* illness and healing’. This analysis of shifting boundaries has resonance for the analysis of gut thinking. In the re-assertion of the importance of matter and physicality (beyond the brain) in research into the gut microbiome’s effects on mental wellbeing, traditional healthcare distinctions between mental and physical health falter. Further boundaries are also called into question, in terms of what constitutes human and non-human, inside and out, and where the lines of health and illness are placed. These lines do not exist separately in different disciplinary spaces but speak to what Fitzgerald and Callard discuss as the ‘deep entanglements of subjectivity, experience, pathology, incorporation, and so on, which cut *across* the ways in which we understand both the human and her medicine today’ [26]. The questions raised by these entanglements are as resonant for biomedicine as they are for humanities scholars and suggest the need for both new methods and vocabularies that outmanoeuvre that Cartesian ‘blind spot’ [14].

### ‘Matter matters’

Critical theorisation in humanities and social sciences in the ‘turn towards the body’ – or ‘the material turn’ – is entangled with the questions raised by microbiome–gut–brain axis research into mental health [49]. Affect theory [50], new materialism [51], feminism [52] and phenomenology [53] have, in different ways, sought to undo the devaluing of the body as unthinking matter. The material turn in humanities scholarship has asked questions about the problematic idea of mind over matter both in individual terms and in relation to the environment. As Stacy Alaimo and Susan Hekman argue, in their scholarship on feminist new materialism, the ‘denigration of nature and the disregard for materiality cannot be entirely disaggregated’ [52]. The dualist conception of mind over matter does more than to denigrate the body; it also positions the human subject as separate from – and superior to – the natural world. Barad’s work, drawn upon by Fitzgerald and Callard [26], helps to redress this, arguing that ‘being is threaded through with mattering’ and, therefore, the nature of materiality itself ‘is an entanglement’ [54]. The challenge from emerging evidence from the gut microbiome counters the mind over matter assumptions of Cartesian medicine, with bodies re-framed as active and relational and comprised of many different genomes of microorganisms, uprooting psych-orientated, individualist, brain-dominant models of behaviour.

### Methods for gut thinking

The concept of the thinking gut may require both biomedicine and humanities disciplines to consider new methods of research. Language tells us that the gut has always been associated with ‘feeling states’ [11] – the notion of ‘gut instinct’ and ‘trusting the gut’ remains present in everyday English language. However, as feminist writer Sara Ahmed explains in her book, *The Cultural Politics of Emotion*, emotional states have also been stratified. Visceral, gut feelings have been characterised as bodily sensations [55]. In healthcare, unreliable bodies have awaited interpretation by the superior, objective, rational mind endorsed by a positivist biomedical epistemology. Subjective and experiential evidence is usually downgraded versus the objective and empirical in terms of medicine’s evidence hierarchies [56]. Healthcare’s bodies do not know, they are to be known. Objectivity is understood to relate to reason and logic, but if the gut is an organ of the mind and the body is involved in thinking, what does this mean for this hierarchy and privileging of mind over body? How might subjective and individual bodies offer insights into minds and guts? Indeed, might what Barad terms as the ‘material practice’ of knowing necessitate an epistemological shift? [57].

If the gut is thinking, it also demands a methodological response from humanities disciplines. Medical humanities as interdisciplinary scholarship has developed a strong focus on applying narrative to questions of medical practice and to the experience of illness [58]. However, ‘embodied methodologies’ [59] that actively look to incorporate body sensation into research may provide scope to connect more specifically with bodies and the sensory nature of ‘mental’ experience and feeling from the gut. These methodologies may range from paying attention to bodies of researchers rather than side-lining them in ‘attempts to eliminate bias’ [60, p. 7], using sensory, physical materials in qualitative interviews with participants, and using bodies in the production or communication of research (walking interviews [61], body mapping [62] or representation in dance, for example) [63]. The gut engages all the senses – from the sound and feel of digestion, to the physical response to the smell, taste or the sight of certain foods or experiences and, as such, requires a sensory, bodily approach to connect with these aspects.

In the final part of this paper, I turn to how the concept of embodiment helps frame evidence from the gut microbiome, moving mental health away from the head and towards bodies intra-acting and entangled with the world, asking what the implications of this are for future research.

## Entangled meanings: what the microbiome–gut–brain axis can contribute to improving understanding about mental health

### Biopsychosocial: horizontal entanglement

Mental health is often framed within a biopsychosocial paradigm [64] – this very definition, despite its emphasis on an integration of perspectives, speaks to disciplinary boundaries of biology, psychology, and social sciences, each taking a vertical disciplinary cut through the mental health conundrum. However, I suggest that the gut microbiome’s evidential links to mood and mental health ask for a much more of a horizontal slice through this biopsychosocial construct. Research into the gut microbiome may be easily divided into the biological research looking at bacteria in the laboratory, with social scientists asking how the social and environmental are related to this new method of internalising mental illness away from social determinants, and psy-disciplines trying to wrestle back selfhood to the head or drawing maps against models of embodied cognition [65]. However, I suggest that it is possible to take a horizontal perspective. This does not mean the eradication of disciplinary boundaries and methodologies but offers an invocation to look differently at the ‘entanglements’ [26] that problematize the vertical linearity from head to body as much as they do the disciplinary silos.

The microbiome–gut–brain axis brings forward a biological basis for mental health problems and gestures towards the social and psychological, not as separate factors, but as enmeshed with the biological. Stress and environmental influences – things that happen to us and shape our lives (even before birth) cause biological changes and responses, and direct expectations of and reactions to future events – not just in terms of thinking from the head, but from within the gut microbiota via ‘long-term modulation of stress-related physiology and behaviour’ [66]. Gut bacteria may be different depending on environment and culture [67] suggesting that cultural and environmental factors are intermingled with bodies, not separate to them. The social is not an externalised force that contributes separately to the bio- and psych-elements, the three are intertwined with one another through the body, and in the world. This way of thinking arguably problematizes the notion of neoliberal selfhood [68] wherein health behaviours (including attempts to improve mental health and wellbeing) are part of a project of self-improvement and empowerment directed from the head [69].

### Gut selfhood: beyond narrative

Research into the microbiome–gut–brain axis has foregrounded an expanded model of selfhood that recognises the influence that intestinal microbes have on cognition and mood. This poses a challenge to what sociologist Rose describes as the ‘regime of the self’, constructed within the ‘psy’ sphere, wherein the physical body is to be known and interpreted by the psyche-brain self in the head [70] (p.3) Furthermore, not only does the gut microbiome’s effect on mood and mental health suggest an undoing of the head over body – mind over matter – understandings about cognition, it has also been argued that it challenges dominant Western philosophical ideas of the boundaried self. Human microbiome research unsettles the idea that human bodies are intact containers, guarding against external invader germs; thus reconceptualising that which may have been previously understood as the non-human, as a part of the human. In a paper on this challenge to selfhood, Rees et al. [71] suggest that evidence showing that microorganisms are a part of human bodies (and the interaction of human and microbial cells) challenges the humanities to re-think what it is to be human away from the ‘untenable’ idea that they might be ‘mere nature’. They argue that a ‘microbial humanities’ is needed to re-think these ‘more than human’ aspects of humanity. This position perhaps risks ignoring the value of existing scholarship that is working to undo nature-culture dualism [26]. However, it does speak to the way in which research on the psychological side of the Cartesian divide has been privileged in medical humanities scholarship on mental health, which has built an emphasis on linguistic and representational issues [72]. Arguably, this is because mental health remains on the side of the Cartesian divide that is linked to speech and language.

The microbiome–gut–brain connection suggests a radical cut through Cartesian boundaries; shifting the emphasis onto the corporeal and challenging the discipline of medical humanities to conceptualise the meaning of matter in relation to mental health. Wilson demonstrates this kind of approach as she argues that in the eating disorder, bulimia nervosa, ‘distress, anger, need, depression, comfort and attachment have become primarily organic’ in nature, such that the division between mood and gut is collapsed [16] (p. 63). The gut responds, conveys and unfolds mood. It is not merely a narrative representation or a metaphor or a secondary somatic response to cognitive thinking. As such, it raises the question of what it means not only to have a body but to be a body, especially when that body is deeply enmeshed with that of microorganisms considered to be non-human.

To move beyond narrative representational enquiry, scholarship on embodiment can be brought towards understanding mental health. In line with phenomenological principles of ‘being in the world’ [53], embodiment goes beyond ‘the body’. As opposed to the static object body (which is managed by the mind), I draw from an interdisciplinary perspective to theorise embodied experience as both proprioceptive (the sense of the relative position of one’s own parts of the body in space) [73] and as that which is always relational, as embodiment scholar Laura L. Ellingson puts it, as ‘mutually constitutive with the world’ [61] (p.3).

## Conclusions: embodied mental health

Mental health is problematic for medicine because it cannot always be seen and shown; organically identified. A model of mental health related to gut dysbiosis moves closer towards medicine’s preferred methods of visual identification. The microbiome–gut–brain axis, like the brain disorder model, seeks to provide a physical, biological location for mental disorder. The very fact that this model has gathered so much attention and interest – with a wave of books and media articles being published since the revival of interest in gut-mind connections and the characterisation of the ‘second brain’ [74] – arguably speaks to the discomfort of conditions that can’t be identified under the microscope of biomedical research. Conditions that rest on subjective feeling are labelled unexplained, functional, or a matter of the intangible mind and, resultantly, are left in an area of problematic stigmatisation. Proponents of alternative models of health may reach to microbiome-gut-brain research to validate why it is necessary to think holistically about health, but data connecting the gut to mood, behaviour and mental health does not provide a neat answer. Additionally, the fact that biomedical evidence frameworks and epistemologies control and constitute what is real (organic) or not – and thereby arguably contribute to stigma – mean that the possibility of such a holistic view is challenged.

Research that suggests the importance of gut health for mental health does not solve the Cartesian traps, especially given the stronghold that language has in shaping perception. However, it does call attention to the possibilities for a model of ‘embodied mental health’ [75], one that recognises that mental health is not a separate entity from physical health and explores the entanglements within a horizontal bio-psycho-social framework. This model reimagines mental health untied from its dualistic roots and unrestricted to the head. It interrogates psychologically driven, individualist constructions of health and wellbeing and demands humanities scholars to think beyond narrative means, to work in much more embodied terms.

For people trying to make sense of mental health problems within ever-shifting models of causation – moving from immaterial thoughts to neuroscience’s brain disorder, to social psychiatry’s focus on social determinants – research into the gut may seem like just another layer of complex explanation. However, such a reversal of head-down, mind over matter culture potentially shifts the focus when it comes to interpreting and making sense of whole-bodied experiences. In an article about anxiety, author, Anna Spargo-Ryan, writes:

It lurches from my throat like a wave of black tar and I choke on it and the world caves in around me and I am drowning. [76]

This is anxiety felt in the ‘very self’ of her body; it is violent and physical and leads to a sense of suffocating enclosure and sensory shutdown. Anxiety is visceral, digestive, figured as surging up, lurching and choking. Anxiety can equally rise from the ground up, from interaction and experience that accumulates and shapes the sense of being a body in the world. Research into the relationship between the gut and the mind does not prove or validate that mental health is beyond the head, but it does entangle the biological, social and psychological. It explores the inseparability of mind and matter and enables a different conceptualisation of mind and the mental to be opened up.
